# The First Cytoplasmic Loop in the Core Structure of the ABCC1 (Multidrug Resistance Protein 1; MRP1) Transporter Contains Multiple Amino Acids Essential for Its Expression

**DOI:** 10.3390/ijms22189710

**Published:** 2021-09-08

**Authors:** Gwenaëlle Conseil, Susan P. C. Cole

**Affiliations:** 1 Division of Cancer Biology and Genetics, Queen’s University Cancer Research Institute, Kingston, ON K7L 3N6, Canada; spc.cole@queensu.ca; 2 Department of Pathology & Molecular Medicine, Queen’s University, Kingston, ON K7L 3N6, Canada

**Keywords:** ABC transporter, cytoplasmic loop, MRP1, multidrug resistance protein, organic anion transport, protein expression, site-directed mutagenesis, structure-guided mutagenesis

## Abstract

ABCC1 (human multidrug resistance protein 1 (hMRP1)) is an ATP-binding cassette transporter which effluxes xeno- and endobiotic organic anions and confers multidrug resistance through active drug efflux. The 17 transmembrane α-helices of hMRP1 are distributed among three membrane spanning domains (MSD0, 1, 2) with MSD1,2 each followed by a nucleotide binding domain to form the 4-domain core structure. Eight conserved residues in the first cytoplasmic loop (CL4) of MSD1 in the descending α-helix (Gly^392^, Tyr^404^, Arg^405^), the perpendicular coupling helix (Asn^412^, Arg^415^, Lys^416^), and the ascending α-helix (Glu^422^, Phe^434^) were targeted for mutagenesis. Mutants with both alanine and same charge substitutions of the coupling helix residues were expressed in HEK cells at wild-type hMRP1 levels and their transport activity was only moderately compromised. In contrast, mutants of the flanking amino acids (G392I, Y404A, R405A/K, E422A/D, and F434Y) were very poorly expressed although Y404F, E422D, and F434A were readily expressed and transport competent. Modeling analyses indicated that Glu^422^ and Arg^615^ could form an ion pair that might stabilize transporter expression. However, this was not supported by exchange mutations E422R/R615E which failed to improve hMRP1 levels. Additional structures accompanied by rigorous biochemical validations are needed to better understand the bonding interactions crucial for stable hMRP1 expression.

## 1. Introduction 

Cellular efflux of many physiological organic anions and pharmacological metabolites is mediated by one or more of the 12 members of the mammalian ATP-binding cassette (ABC) (subfamily C) (ABCC) family of membrane proteins [1,2]. Eight of the ABCC proteins are collectively referred to as the multidrug resistance proteins (MRPs), the first of which, human MRP1/ABCC1 (hMRP1), was identified based on its ability to confer resistance to natural product antineoplastic agents such as doxorubicin and vincristine [3,4,5,6]. Since then, hMRP1 has been shown to also efflux a diverse array of conjugated xenobiotic and physiologic organic anions [4,5]. Foremost among the latter are the proinflammatory cysteinyl leukotriene C_4_ (LTC_4_) [7] and the conjugated estrogens, estradiol glucuronide (E_2_17βG) and estrone sulfate (E_1_3SO_4_) [8,9].

**Figure 1:**
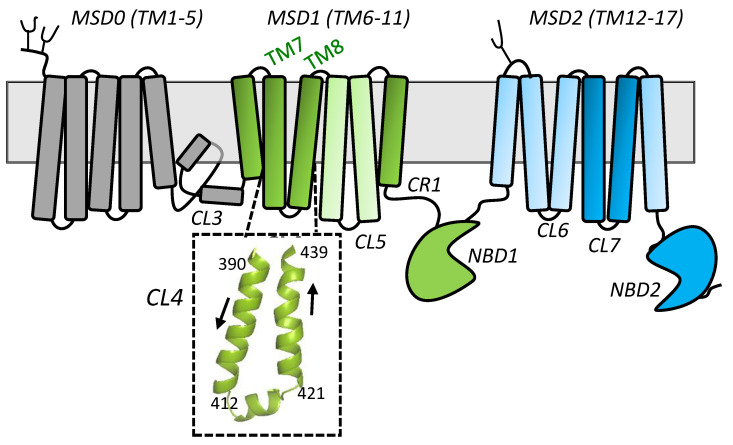
Schematic diagram of the secondary structure of human MRP1 highlighting CL4. Shown is a linear 2-dimensional illustration of the domain structure of MRP1/ABCC1. MSD0 (TM 1-5) (gray) is present only in a subset of 8 ABCC/MRP subfamily members and is connected to MSD1 (TM 6-11) by cytoplasmic loop (CL) 3 (also often referred to as L_0_). The MRP1/ABCC1 core structure is comprised of MSD1/NBD1 (green), and MSD2/NBD2 (blue). In its native 3-dimensional state, the 12 TM of MSD1 and MSD2 are assembled into 2 helical bundles: helix bundle 1 consisting of TM 6, 7, 8, 11, 15, 16 (dark green/blue) and helix bundle 2 consisting of TM 9, 10, 12, 13, 14, 17 (light green/blue). CL4 links TM7 to TM8 in MSD1 and is enlarged to show its 3-dimensional structure based on an atomic homology model of the hMRP1 core structure generated using the cryogenic electron microscopic (cryo-EM) structure of apo bovine Mrp1/Abcc1 (bMrp1/Abcc1) (PDB: 5UJ9) as template [14]. CL, cytoplasmic loop; CR1, connecting region 1; MSD, membrane spanning domain; NBD, nucleotide binding domain; TM, transmembrane.

The 190 kDa hMRP1 contains 17 transmembrane (TM) α-helices distributed over three membrane spanning domains (MSD0, 1, 2) with a nucleotide binding domain (NBD1, 2) following each of MSD1 and MSD2 which together comprise the 4-domain core structure of the protein (MSD1-NBD1-MSD2-NBD2) (Figure 1). The Walker A and B motifs of NBD2 and the ‘C’ signature motif of NBD1 of MRP1 form a consensus nucleotide binding site that can bind and hydrolyze ATP. In contrast, the Walker A and B motifs of NBD1 and the ‘C’ signature motif of NBD2 form a non-consensus, so-called ‘degenerate’ nucleotide binding site which binds but only poorly hydrolyzes ATP [10]. This degenerate site is characteristic of all ABCC subfamily members and a subset of ABCB subfamily members [11]. The processes involved in the intradomain folding and interdomain assembly of the five domains of MRP1 into its native, transport-competent conformation, like other polytopic ABC membrane proteins, are complex and not yet well understood [11,12,13].

One experimental strategy employed extensively to identify amino acids important for proper expression and function of hMRP1 is site-directed mutagenesis. Using this approach, the cytoplasmic loops (CLs) between adjacent TMs in the core MSD1 and MSD2 have been shown in several instances to serve a function more important than simply connecting their respective TMs. For example, we have reported previously that uncharged alanine substitutions of ionizable residues Lys^513^, Lys^516^, Glu^521^, and Glu^535^ in CL5 (which links TM9 to TM10) [15] and Arg^1166^ and Asp^1183^ in CL7 (which links TM15 to TM16) [16] result in a failure of hMRP1 protein to be expressed in transfected mammalian (HEK) cells. In almost all cases (CL7-D1183E is thus far the sole exception [16]), mutants with same charge substitutions of these highly conserved residues are expressed and active. These findings have established that the specific geometries of the side chains of charged residues within these cytoplasmic regions of hMRP1 are important for the proper folding and ultimately plasma membrane localization of the transporter [12].

A substantial body of evidence indicates that a crucial step in the intradomain folding and interdomain assembly of ABC exporters that ensures their proper expression and membrane trafficking requires the engagement of the second CL of each MSD into a ‘hollow’ in the opposite NBD (in the case of hMRP1, MSD1-CL5, and MSD2-CL7 into NBD2 and NBD1, respectively). These processes are thought to be stabilized by charge interactions in conserved positions [11,12,13]. Previous studies demonstrating the importance of charged amino acids in CL5 and CL7 [15,16], as well as residues in NBD1 [17,18] and NBD2 [15], of hMRP1 support this requirement. However, mutation of amino acids in other regions of hMRP1 also result in loss of protein expression. For example, in a recent study, we identified three amino acids (Arg^615^, Phe^619^, Glu^624^) in the cytoplasmic connecting region (CR1) that links TM11 to NBD1 (Figure 1) as being essential for hMRP1 expression in mammalian cells [19]. Based on potential electrostatic bonding interactions of Arg^615^, Phe^619^, and Glu^624^ with nearby amino acids (as suggested by the cryo-EM structure of apo bMrp1/Abcc1 [20] as well as two homology models of apo hMRP1 [14,21]), we used reciprocal exchange mutagenesis as a means of testing whether maintaining the putative bonding interactions would restore or improve hMRP1 expression. However, none of the three exchange mutant proteins investigated (R615D/D430R, R615F/F619R, E624K/K406E) were detectable by standard immunoblotting. Nevertheless, these studies revealed that both non-conservative and charge preserving substitutions of Lys^406^ in CL4 (a stretch of approximately 50 amino acids that links TM7 to TM8) (Figure 1) caused a loss of hMRP1 expression [19].

Our finding that CL4-Lys^406^ was crucial for hMRP1 expression was somewhat unexpected because we previously showed that non-conservative substitutions of only one (Asp^430^) of five (i.e., Arg^394^, Lys^396^, Arg^433^, and Asp^436^) charged residues in CL4 had any adverse effect on hMRP1 levels [22,23]. In addition, cryo-EM studies of ATP-bound E1454Q mutant bMrp1/Abcc1 (PDB: 6BHU) indicate potential bonding interactions between CL4 residues and an ATP molecule captured at the degenerate nucleotide binding site [11,24]. Together, these studies made it seem more likely that CL4 mutations might have a deleterious effect on hMRP1 function rather than on its expression. Consequently, our observations that mutations of both Lys^406^ and Asp^430^ abrogated hMRP1 expression in transfected HEK cells have prompted us to further characterize CL4. Here, we have used primary sequence alignments as well as available cryo-EM structures and hMRP1 homology models to select additional residues in this cytoplasmic region (defined here as amino acids 390–439) to target for mutagenesis. We then investigated the effects of the targeted CL4 mutations on hMRP1 levels and when possible, their transport function after expression in HEK cells.

## 2. Results

### 2.1. Sequence Alignments and Initial Selection of CL4 Amino Acids for Mutagenesis

**Figure 2:**
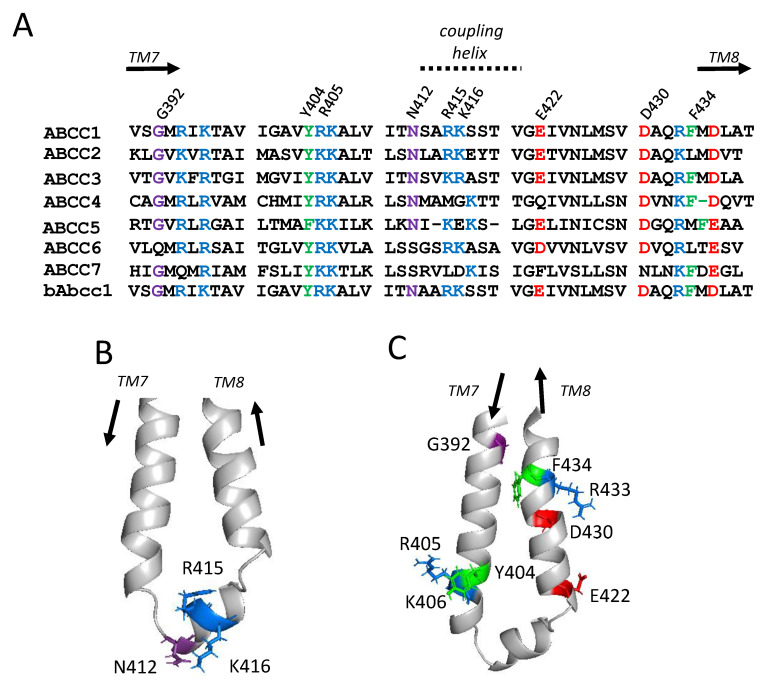
Sequence alignments of hMRP1 CL4 and location of residues targeted for mutagenesis. (**A**) Shown is a sequence alignment of CL4 of hMRP1/ABCC1 with the corresponding region of six of its human homologs (ABCC2–7) and bMrp1/Abcc1 generated using Clustal Omega [26]. The basic (Arg^405^, Arg^415^, Lys^416^), acidic (Glu^422^) and aromatic (Tyr^404^, Phe^434^) amino acids targeted in the present study are highlighted in blue, red, and green, respectively, as are the corresponding residues (if conserved) in human ABCC2–7 and bMrp1/Abcc1. The targeted Gly^392^ and Asn^412^ are also highlighted (in purple) are the targeted Gly^392^ and Asn^412^. Basic (Arg^394^, Lys^396^, Lys^406^, Arg^433^) and acidic (Asp^430^, Asp^436^) hMRP1 CL4 residues mutated in previous studies [19,22,23] are also highlighted in blue and red, respectively. (**B**,**C**) Shown are the locations of the targeted residues within a selected view of a 3-dimensional atomic homology model of hMRP1-CL4 [12] with their side chains shown in stick form and colored as in (**A**); (**B**) Asn^412^, Arg^415^ and Lys^416^ in the coupling helix of CL4; (**C**) Gly^392^, Tyr^404^, Arg^405^, Lys^406^, Glu^422^, Asp^430^, Arg^433^, and Phe^434^ outside (‘flanking’) the coupling helix.

To determine which CL4 residues to target for substitution, multiple sequences of hMRP1 amino acids 390-439 with its human homologs ABCC2-ABCC7 (MRP2-5, ABCC6, CFTR; P33527, Q92887, O15438, O15439, O15440, O95255, P13569) were retrieved from UniProt [25] and alignments generated using Clustal Omega [26] [www.ebi.ac.uk/Tools/msa/clustalo/; accessed on 1 March 2021]. These sequences were reviewed together with the comparable region of bMrp1/Abcc1 (Q8HXQ5) because this hMRP1 ortholog has been used in all cryo-EM studies published to date (Figure 2A) [20,24].

CL4 is comprised of three segments beginning with an upstream descending cytoplasmic α-helical extension of TM7 followed by a coupling helix region which is roughly parallel to the plasma membrane [11,12,24]. The coupling helix is then followed by an ascending α-helical stretch that re-inserts back into the membrane as TM8 (Figure 1). Based on earlier studies demonstrating the importance of conserved charged residues in the CLs for hMRP1 expression in mammalian cells [15,16], the highly conserved Arg^415^ and Lys^416^ from the CL4 coupling helix (amino acids 412–421) were first selected for analysis (Figure 2B). Asn^412^ from this region was also included because this highly conserved polar residue is reported to interact with the bound ATP molecule at the degenerate nucleotide binding site referred to earlier [11,24].

We have reported previously that opposite charge substitutions of the highly conserved Arg^394^ and Lys^396^ in the cytoplasmic helical extension of TM7 upstream of the CL4 coupling helix as well as the downstream Arg^433^ and Asp^436^ in the α-helical extension preceding TM8 had no effect on hMRP1 expression levels [22,23]. In contrast, substitutions of the upstream Lys^406^ and the downstream Asp^430^ resulted in complete loss of detectable hMRP1 [19,23]. For this reason, we also selected the highly conserved upstream Arg^405^ and downstream Glu^422^ for mutagenesis (Figure 2C). In addition, because of our recent unexpected observation that Phe^619^ in CR1 was sensitive to mutation and crucial for hMRP1 expression [19], the highly conserved aromatic Tyr^404^ and Phe^434^ upstream and downstream of the CL4 coupling helix, respectively, were targeted for mutagenesis as well (Figure 2C). The final CL4 residue selected for mutagenesis was the highly conserved upstream Gly^392^ located at the juxtaposition of CL4 with TM7 at the inner leaflet of the plasma membrane (Figure 2C).

### 2.2. Effect of Mutating Asn^412^, Arg^415^, and Lys^416^ in the Coupling Helix of CL4 

In the first series of experiments, alanine substitutions of coupling helix Asn^412^, Arg^415^, and Lys^416^ and same charge substitutions of Arg^415^ and Lys^416^ were generated by site-directed mutagenesis, and the mutant hMRP1 constructs were transiently transfected into HEK cells. After 48 h, no adverse effects on the growth or viability of the transfected cells were observed. The cells were then collected, whole cell detergent extracts (WCE) prepared and total hMRP1 levels estimated by immunoblotting and densitometry. As shown in Table 1, all five of the CL4 coupling helix mutant proteins (N412A, R415A, R415K, K416A, and K416R) were readily detectable at levels comparable to wild-type hMRP1 (83% to 138% of wild-type hMRP1). These observations indicate that preserving these residues (or their charge) in the coupling helix segment of CL4 is not essential for hMRP1 expression in HEK cells.

**Figure 3:**
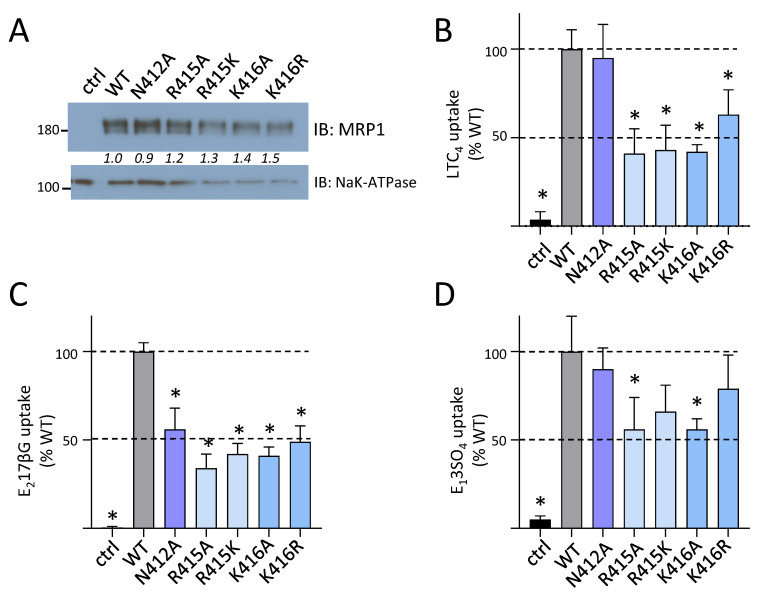
Effect of CL4 coupling helix mutations N412A, R415A/K and K416A/R on hMRP1 organic anion transport activity. (**A**) Shown is a representative immunoblot (2 s exposure) of inside-out membrane vesicles (1 µg protein per lane) prepared from HEK cells transfected with wild-type (WT) and N412A, R415A/K and K416A/R mutant hMRP1 pcDNA3.1 expression vectors as well as untransfected HEK cells (ctrl). Blots were probed with anti-hMRP1 (Mab QCRL-1) and anti-Na^+^/K^+^-ATPase as a membrane protein loading control; the region between hMRP1 and Na^+^/K^+^-ATPase signals has been cropped out. Molecular weight markers (kDa) are to the left. Italicized numbers between the 2 panels indicate mutant hMRP1 levels relative to wild-type hMRP1 after correcting for levels of the protein loading control as measured by densitometry. (**B**–**D**) Transport activity was measured as ATP-dependent uptake of (**B**) [^3^H]LTC_4_, (**C**) [^3^H]E_2_17βG and (**D**) [^3^H]E_1_3SO_4_ (in the presence of 3 mM S-methyl GSH) into inside-out membrane vesicles prepared from transfected cells and expressed as a percent of uptake by wild-type hMRP1. The values shown have been adjusted to take into account minor differences in mutant hMRP1 levels in the membrane vesicles relative to wild-type (WT) hMRP1. Bars represent the mean values (±SD) of results obtained from three independent experiments. * Significantly different from wild-type hMRP1 (*p* < 0.05).

Given that levels of the CL4 coupling helix mutants N412A, R415A/K, K416A/R were comparable to those of wild-type hMRP1, we next determined if any of the mutations affected hMRP1 transport function. Accordingly, inside-out membrane vesicles were prepared from transfected HEK cells expressing the five mutant hMRP1 proteins and immunoblotting showed that membrane levels of the N412A, R415A/K, and K416A/R mutants, as observed for WCE, were comparable to wild-type hMRP1 (81% to 127% of wild-type hMRP1 levels; Table 1) (Figure 3A). Subsequent transport assays showed that levels of ATP-dependent [^3^H]LTC_4_ [7] (Figure 3B), [^3^H]E_2_17βG [15] (Figure 3C), and S-Me GSH stimulated [^3^H]E_1_3SO_4_ [8,9] (Figure 3D) uptake into inside-out membrane vesicles enriched for the coupling helix mutants was moderately but significantly reduced (to approximately 40–60% of wild-type hMRP1 levels) for at least one but more often, all three organic anion substrates tested (Figure 3B–D). The least affected coupling helix mutant was N412A, which exhibited a selective and moderate (50%) decrease in E_2_17βG transport but retained wild-type levels of LTC_4_ and S-methyl GSH stimulated E_1_3SO_4_ transport. In comparison, both non-conservative (Ala) and conservative (same charge) mutations of Arg^415^ and Lys^416^ almost always resulted in moderate (40–60%) and significant (*p* < 0.05) decreases in transport of all three organic anions. Taken together, these data indicate that while both alanine and conservative substitutions of these three CL4 coupling helix residues have no deleterious effect on hMRP1 levels, all of the mutations caused a moderate but significant decrease in the ATP-dependent transport of at least one, if not all of the three organic anions tested.

### 2.3. Effect of Mutating Gly^392^, Tyr^404^, and Arg^405^ Upstream and Glu^422^ and Phe^434^ Downstream of the CL4 Coupling Helix on hMRP1 

**Figure 4:**
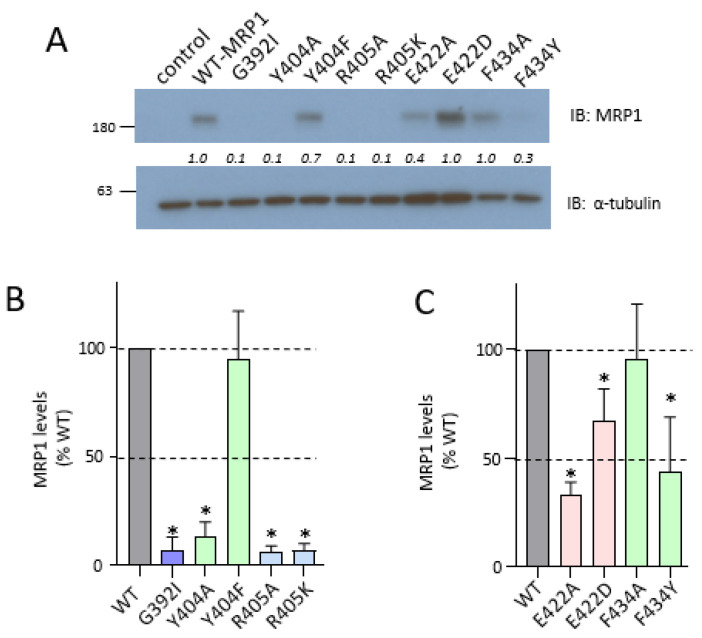
Effect of mutating residues outside the CL4 coupling helix on hMRP1 protein expression levels in HEK cells. Wild-type and mutant hMRP1 expression levels were measured by immunoblotting of WCE prepared from transfected HEK cells. Blots were probed with anti-hMRP1 (Mab QCRL-1) and anti-α-tubulin as a protein loading control. (**A**) Representative immunoblot of WCE (10 µg per lane) prepared from all upstream (G392I, Y404A, Y404F, R405A, R405K) and downstream (E422A, E422D, F434A, F434Y) mutants. Relative levels of wild-type (WT) and mutant hMRP1 were estimated using densitometry, and values were normalized according to the α-tubulin signal (shown in italics). The region between the hMRP1 and the α-tubulin signals has been cropped out; molecular weight markers (kDa) are indicated on the left of the blot. (**B**,**C**) Quantitation of mutant hMRP1 levels, with each bar representing the mean (±SD) of results obtained from 3–5 independent transfections and expressed as a percent of wild-type (WT) hMRP1 levels: (**B**) upstream mutants (G392I, Y404A, Y404F, R405A, R405K); (**C**) downstream mutants (E422A, E422D, F434A, F434Y). * Significantly different from wild-type hMRP1 (*p* < 0.05).

In the next series of experiments, additional amino acids flanking the CL4 coupling helix were targeted. We have previously shown that the non-conservative Ala-substituted and opposite charge mutants of the highly conserved downstream Asp^430^ (D430A; D430R; D430K) were very poorly expressed whereas levels of the same charge D430E mutant were comparable to wild-type hMRP1 [19,23] as were levels of the R433S mutant corresponding to a low frequency non-synonymous single nucleotide polymorphism of *ABCC1* [22]. In contrast, both the Ala-substituted as well as the same charge mutants of the highly conserved upstream Lys^406^ (K406A; K406R) were poorly expressed [19]. In view of these differences, we generated both non-conservative alanine and conservative substitutions of Tyr^404^, Arg^405^, Glu^422^, and Phe^434^; in addition, a bulky isoleucine substitution of Gly^392^ (Figure 2C) was created. The mutant hMRP1 constructs were then transfected into HEK cells, WCE prepared and immunoblotted as above. As shown in Figure 4A,B, four of five of the upstream mutant proteins (G392I, Y404A, R405A, and R405K), like the K406A/E/R mutants [19], were barely detectable. The single exception was the conservatively substituted Y404F which was expressed at levels not significantly different than wild-type hMRP1 (95% ± 22%; *p* > 0.05). In contrast, all four of the downstream mutants (E422A, E422D, F434A, and F434Y), like the same charge D430E [19], were readily detected although levels were moderately decreased compared to the wild-type transporter in three of four cases (Figure 4A,C). Thus, E422A levels were significantly lower than wild-type hMRP1 (just 33% ± 6%; *p* < 0.05) whereas those of the conservatively substituted E422D were closer to wild-type hMRP1 levels (67% ± 15%; *p* < 0.05). Levels of the non-conservatively substituted F434A were also comparable to wild-type hMRP1 (96% ± 25%; *p* > 0.05) but those of the more conservatively substituted F434Y were significantly lower (just 44% ± 25% of wild-type; *p* < 0.05). Although levels of the latter mutant were unusually variable, these observations indicate that the introduction of the polar aromatic Tyr at position 434 in CL4 had a moderately deleterious effect on hMRP1 expression levels.

Given that levels of the conservatively substituted CL4 upstream Y404F mutant and the downstream E422D and D430E mutants were comparable or close to wild-type hMRP1, we next determined if these mutants also retained their organic anion transport activity. Inside-out membrane vesicles were prepared from transfected cells, immunoblotted (Figure S1A) and ATP-dependent uptake of [^3^H]LTC_4_, [^3^H]E_2_17βG and [^3^H]E_1_3SO_4_ in the presence of S-methyl GSH was measured as before (Figure S1B–D). As summarized in Table 2, the transport activities of all three conservatively substituted mutants (Y404F, E422D, D430E) were comparable to wild-type MRP1 (*p* > 0.05), regardless which of the three organic anion substrates was tested.

### 2.4. Effect of Exchange Mutations of CL4-Glu^422^ and CR1-Arg^615^ on hMRP1 Levels in HEK Cells

In the last set of experiments, the ‘measurement’ tool of the molecular visualization program PyMOL was used to examine apo bMrp1/Abcc1 (PDB: 5UJ9) [20], and two atomic homology models of apo hMRP1: one based on the cryo-EM structure of apo bMrp1/Abcc1 (PDB: 5UJ9) [14] and the other based on the crystal structure of nucleotide-free apo TM287/288 from *T. maritima* (PDB: 4Q4H) [21]. The aim was to determine if they might suggest the existence of electrostatic bonding interactions (salt bridges or other ionic interactions) of the mutation-sensitive ionizable Glu^422^ and Arg^405^ with nearby amino acids that could help explain why neutral or non-conservative substitutions of these residues resulted in poorly expressed mutant hMRP1 proteins. 

**Figure 5:**
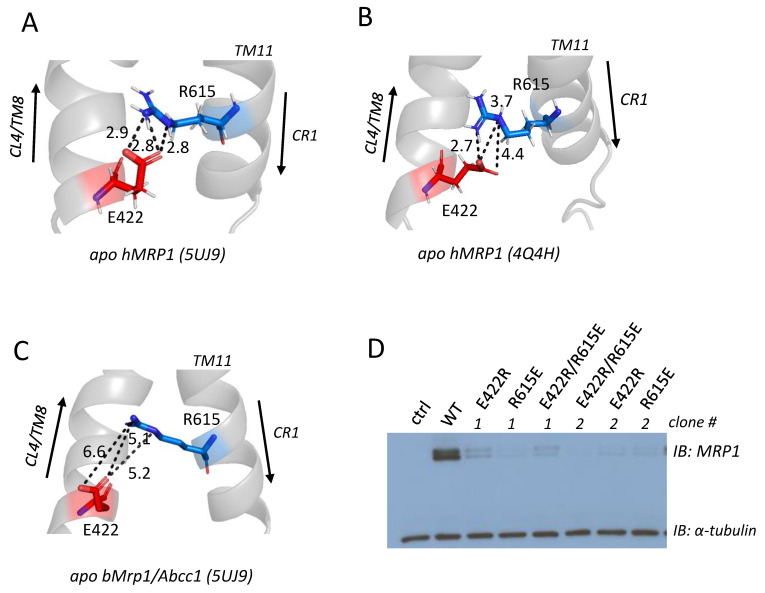
Potential bonding interactions of CL4-Glu^422^ and effect of double exchange mutation with CR1-Arg^615^ on hMRP1 levels in HEK cells. Shown are three views of the potential electrostatic bonding interactions of the CL4-Glu^422^ (red) acidic side chain with the basic side chain of Arg^615^ (blue) located in CR1 (between TM11 and NBD1) observed in (**A**,**B**) the atomic homology models of hMRP1 based on (**A**) the apo bMrp1/Abcc1 cryo-EM structure (PDB: 5UJ9) [14] and (**B**) the apo TM287/288 crystal structure (PDB: 4Q4H) [21]. (**C**) in the apo bMrp1/Abcc1 cryo-EM structure (PDB: 5UJ9) [20]. The distances (in Å) between selected ion pairs (dotted lines) are indicated and were determined using the measuring tool of PyMOL. For further details, see Table S1. (**D**) Shown is a representative 5 s exposure immunoblot of WCE (10 µg protein per lane) prepared from HEK cells transfected with expression vectors encoding wild-type (WT) and two independent clones (# 1 & 2) of mutant (E422R, R615E, and E422R/R615E) hMRP1. A WCE of untransfected HEK cells served as a negative control (ctrl). hMRP1 was detected with Mab QCRL-1 and anti-α-tubulin was used as a protein loading control.

In the apo bMrp1/Abcc1 cryo-EM structure and the apo hMRP1 homology model based on it, a plausible salt bridge between the side chains of Arg^405^ and Asp^1179^ was identified (Table S1) (i.e., with predicted interatomic distances between the charged centers of contributing atoms ≤4Å) [27,28]. However, we previously showed that a non-charged alanine substitution of Asp^1179^ had no deleterious effect on hMRP1 levels [16], indicating that any electrostatic interactions that might exist between Asp^1179^ and Arg^405^ are unlikely to be crucial for stable hMRP1 expression. It is worth noting that in the hMRP1 model based on the apo TM287/288 crystal structure (PDB: 4Q4H) [21], no electrostatic bonding interactions of the ionizable Arg^405^ side chain with any nearby amino acids were detected (i.e., all distances between centers of charge or polarity are substantially >4Å (7.1–11.3Å)) (Table S1). In the case of Glu^422^, however, both apo hMRP1 models (Figure 5A,B) (but not apo bMrp1/Abcc1) (Figure 5C) predict that the oxygen atoms of the CL4-Glu^422^ γ-carboxylate group and the cationic guanidinium group of the Arg^615^ side chain in CR1 are within 4Å of each other (Table S1), supporting the possibility of a salt bridge and extensive hydrogen bonding between these residues [28]. Consequently, the reciprocal charge exchange mutants of CR1-Arg^615^ and CL4-Glu^422^ that might be expected to preserve the electrostatic bonds, and therefore restore or at least improve MRP1 expression levels [29,30,31], were generated by mutagenesis, two independent clones expressed in HEK cells, and WCE prepared and immunoblotted as before. As shown in Figure 5D, both clones of the double exchange mutant E422R/R615E and its corresponding E422R and R615E single mutant controls (like the Ala-substituted E422A and R615A) [19], were all expressed very poorly (levels <5% wild-type hMRP1) and barely detectable in immunoblots even after a prolonged film exposure time (up to 30 s).

## 3. Discussion

It is now widely accepted that amino acids within the CLs that link the TM helices of ABC proteins such as hMRP1 can be of both functional and structural importance. For hMRP1, most studies to date have focused on the second CL in MSD1 (CL5) and MSD2 (CL7) because both biochemical investigations and structures of related ABC proteins indicate that they form close contacts with the two nucleotide binding sites of the transporter [11,12,13,14,15,19,32]. However, the first CL of the core MSD1,2 of hMRP1 (CL4, CL6) and several other ABC proteins have also been shown to play important roles in their structures and/or functions [19,23,33,34,35,36,37]. Here we have explored further the role(s) of CL4 of hMRP1 including the coupling helix at the nadir of the loop as well as its flanking upstream descending and downstream ascending cytoplasmic α-helical sequences by targeted mutagenesis. The results presented, together with those of our previous studies [19,23], demonstrate that amino acids flanking the CL4 coupling helix, rather than residues within the coupling helix itself, are crucial for proper expression of the hMRP1 transporter.

It has been proposed previously that the coupling helix of the first CL in MSD1 of ABC transporters has a functional role in coupling the nucleotide occupancy of the first nucleotide binding site with the orientation of the TM helices [11,13,34]. Indeed, the cryo-EM map of ATP-bound E1454Q mutant bMrp1/Abcc1 (PDB: 4BHU) indicates that the adenine moiety of the ATP molecule in the degenerate nucleotide binding site is proximal to Asn^412^ and Lys^416^ [24]. Amino acids in the hMRP1 CL4 coupling helix are highly conserved and contain at least one, and more often two adjacent, basic residues (Figure 2A). We found that an uncharged, cavity-creating alanine substitution of either Arg^415^ or Lys^416^ had no effect on hMRP1 expression levels indicating that neither of these residues are essential for the proper folding and assembly of the transporter into a stable conformation (Figure 3). On the other hand, these substitutions had a moderately adverse effect (approximately 50% decrease) on the transport of three different organic anions by hMRP1. A similar non-substrate-selective decrease in transport activity was observed when either of these two adjacent basic residues in hMRP1 was replaced with a same charge amino acid. These observations indicate that preserving the positive charges of the Arg^415^ and Lys^416^ side chains in the CL4 coupling helix is not sufficient for hMRP1 to retain its full transport activity and therefore the distinct physicochemical properties of the different adjacent basic side chains (and their potential influence on each other) must play a role. 

Alanine substitution of the polar Asn^412^ in the CL4 coupling helix also had no adverse effect on hMRP1 expression levels and even less of an effect on organic anion transport than mutation of either Arg^415^ or Lys^416^ (Figure 3). Together, these results support the conclusion that none of these three coupling helix residues are essential for hMRP1 expression and further, are only moderately important for its transport activity. The latter observations are somewhat surprising since it was anticipated that the loss of any one of these residues would disrupt the geometry of (and therefore ATP binding at) the degenerate nucleotide binding site [24]. Because formation of a proper interface between the first CL of MSD1 and NBD1 is essential for ABC transporter (or channel) function [11,33,34,35,36], it may be concluded that, despite their relatively high conservation, none of these three amino acids are involved, at least in hMRP1. Of interest however, is the report that a variant of the hMRP1 homolog hABCC6 (p.Ser398Arg) analogous to hMRP1-Asn^412^ is associated with the hereditary mineralization disorder known as pseudoxanthoma elasticum [37]. 

Flanking the CL4 coupling helix, Lys^406^ (upstream) and Asp^430^ (downstream) have been previously identified as crucial for the expression of hMRP1 rather than its function [19,23]. In the present study, we have expanded the ‘inventory’ of CL4 amino acids important for hMRP1 expression by demonstrating that non-conservative substitutions of the upstream Gly^392^, Tyr^404^, Arg^405^ and downstream Glu^422^ substantially or completely abrogated hMRP1 levels in HEK cells (Figure 4). The role of the highly conserved Gly^392^ proximal to TM7 in hMRP1 expression is not known but is likely related to its rotational freedom due to its lack of a side chain because its replacement with a bulky, sterically constrained, hydrophobic isoleucine was not tolerated. This may be simply because the isoleucine side chain distorts this descending α-helical segment of CL4 thus disrupting the overall geometry of the loop (and therefore any stabilizing interdomain interactions of MSD1). Alternatively, given the relatively close proximity of Gly^392^ to the ascending α-helical segment of CL4 at the juxtaposition of TM8 at the plasma membrane (Figure 2C), the bulkier isoleucine may somehow indirectly (through an inter-helical action), impair the re-entry of this CL into the membrane during biogenesis. Regardless of the explanation, hMRP1 protein expression has clearly been disrupted by the loss of this glycine residue. It is worth noting that a disease-associated non-conservative substitution of the analogous Gly^149^ (p.Gly149Arg) in ABCC7 (better known as the cystic fibrosis transmembrane conductance regulator or CFTR) also resulted in barely detectable levels of this chloride channel [33,34] suggesting a conserved role for this amino acid. 

With respect to Tyr^404^ and Arg^405^, we note that these two residues, together with the previously described mutation-sensitive Lys^406^ [19], form a highly conserved polar ‘triad’ in the upstream α-helical segment of CL4 (Figure 2A). The fact that the like-charged Arg^405^ and Lys^406^ are adjacent to one another likely introduces some significant geometrical and electrostatic constraints on this CL4 segment due to some degree of charge repulsion which in turn may help stabilize its position as it descends from the plasma membrane [27]. In our previous study, we observed that not only was the cavity-creating, non-conservative K406A mutant poorly expressed but so too was the conservative same charge K406R mutant [19]. Similarly in the present study, mutants with an alanine or same charge substitution of the adjacent Arg^405^ were poorly expressed (Figure 4A). Together, these results indicate that while positive charges at positions 405 and 406 are important for hMRP1 expression, they are not sufficient and thus the distinctive volumes, geometry and electrostatic properties of the ionizable Arg^405^ and Lys^406^ side chains also play some more nuanced role in hMRP1 biogenesis. Of potential relevance is the observation that a variant of the hMRP1 homolog hABCC6 (p.Arg391Gly) corresponding to hMRP1-Arg^405^ is associated with a mild, late onset form of pseudoxanthoma elasticum [38]. 

In contrast to the poorly expressed, same charge upstream mutants R405K and K406R (and non-conserved Y404A), the conservatively substituted Y404F was expressed at wild-type hMRP1 levels (Figure 4A). This indicates that the loss of a polar substituent at position 404 does not affect the role of this amino acid in promoting stable hMRP1 expression as long as aromaticity is preserved. In addition, Y404F exhibited wild-type levels of organic anion transport activity (Table 2; Figure S1A–D). The observation that only the aromaticity of Tyr^404^ is crucial suggests that its side chain might be involved in stabilizing π-π stacking and/or π-cation interactions with nearby aromatic or positively charged amino acids, respectively [39,40], at least at some point during biogenesis and/or assembly of hMRP1. However, such interactions are not apparent in any of the current models of hMRP1 or bAbcc1/Mrp1, and thus precisely how the upstream Tyr^404^ promotes stable hMRP1 expression remains to be determined. 

Mutations of Glu^422^, Asp^430^ and Phe^434^ downstream of the CL4 coupling helix appear much less disruptive to hMRP1 expression levels than the upstream mutations. Whereas we showed previously that non-conservative substitutions of Asp^430^ abrogated hMRP1 expression, the same charge D430E is expressed at wild-type levels [19,23]. Similarly, alanine substitution of Glu^422^ reduced hMRP1 expression to about 35% of wild-type levels but when replaced by a same charge aspartate, expression improved to 65% of wild-type hMRP1 levels (Figure 4B). This suggests that while a negative charge is important at both positions 422 and 430, the distinctive geometries and electrostatic properties of the glutamate and aspartate side chains play a lesser role. Moreover, unlike the non-expressed upstream R405K and K406R mutants, not only were the same charge downstream mutants E422D and D430E readily expressed, they also exhibited organic anion transport activities comparable to wild-type hMRP1 (Table 2; Figure S1). These results indicate that whatever stabilizing interactions the latter charged amino acids form in the biogenesis of native, transport-competent hMRP1, they can be maintained (or mostly maintained) as long as the charges of their side chains are preserved, even if their other distinctive physicochemical properties are not. 

In contrast to Glu^422^ and Asp^430^, alanine substitution of the highly conserved Phe^434^ did not adversely affect hMRP1 expression levels showing that, unlike Tyr^404^ in the upstream α-helical segment of CL4, the aromatic side chain of this downstream residue is not crucial for hMRP1 expression. On the other hand (and in contrast to Y404F as well as E422D and D430E), introduction of a conservative but polar aromatic Tyr at position 434 (F434Y) caused a significant (50%) decrease in hMRP1 levels (Figure 4B). Thus, the presence of a tyrosine with a polar hydroxyl group at position 434 appears to be moderately disruptive. It is worth noting that prior to this study, only two aromatic residues had been identified as being important for hMRP1 expression, viz., Phe^728^ in NBD1 (A. Hernandez, S.P.C. Cole, unpublished) and Phe^619^ in CR1 [19]. hMRP1 Phe^619^ and Phe^728^ correspond to Phe^374^ and Phe^508^ in CFTR/ABCC7, respectively, which have also been demonstrated to be critical for the stable expression of this gated chloride channel indicating a conserved function [41,42]. In the present study, hMRP1-Phe^434^ corresponds to CFTR-Phe^191^ and a mutation (p.Phe191Leu) has been detected in a patient with cystic fibrosis [43]. To the best of our knowledge, however, no functional studies of this CFTR/ABCC7 mutation have been reported. 

In silico analyses of the apo bMrp1/Abcc1 cryo-EM structure and two homology models of apo hMRP1 of the atomic environment of the most mutation-sensitive polar/ionizable CL4 residues (i.e., Tyr^404^, Arg^405^, Lys^406^, Glu^422^, Asp^430^) yielded relatively little information about potentially stabilizing electrostatic bonding interactions of their side chains [14,20,21]. For Lys^406^ and Asp^430^ (as noted earlier), reciprocal exchange mutagenesis with CR1-Glu^624^ and CR1-Arg^615^, respectively, to restore predicted ionic interactions failed to improve hMRP1 expression levels above the very low levels observed for the individual single mutants [19]. Further, as noted earlier, the models do not reveal any potential bonding partners for Tyr^404^. For Arg^405^, stabilizing interactions with Asp^1179^ (Table S1) seem highly unlikely because alanine substitution of this CL7 residue had no effect on hMRP1 levels [16]. For Glu^422^, however, we identified several potential electrostatic interactions of its γ-carboxylate moiety with CR1-Arg^615^ which, unlike the ‘buried’ Arg^405^/Asp^1179^ ion pair, are facing into the aqueous pore of hMRP1 and therefore considered more energetically favorable (Figure 5A,B) (Table S1) [44]. Disappointingly, we found that levels of the E422R/R615E exchange mutant nevertheless remained >80% lower than wild-type hMRP1 and comparable to the poorly expressed single mutants E422A, E422R, R615E, and R615A (Figure 5D) [19]. There are several reasons why these results do not necessarily preclude the existence of electrostatic bonding interactions between CL4-Glu^422^ and CR1-Arg^615^, including the simple possibility that ion pairing between these residues is not involved in promoting stability of the folded protein [44]. It is also possible that the targeted residues participate in multiple critical inter- and/or intra-helical bonding interactions that are not restored when the two residues are exchanged. Relevant to this suggestion, further consideration of the hMRP1 atomic homology models revealed the close proximity of the Asp^430^, Arg^615^, and Glu^422^ side chains to one another and thus the possibility of a more complex network of stabilizing ionic and hydrogen bonds among the three residues that would not have been restored in the double mutants (Figure S2A,B). 

## 4. Materials and Methods

### 4.1. Materials

[6,7-^3^H]E_2_17βG (46.3 Ci mmol^−1^) (NET1106), [6,7-^3^H]E_1_3SO_4_ (51.8 Ci mmol^−1^) (NET203) and [14,15,19,20-^3^H]LTC_4_ (170.2 Ci mmol^−1^) (NET1018) were from Perkin Elmer Life Sciences (Boston, MA, USA). LTC_4_ (20210) was from Cayman Chemical (Ann Arbor, MI, USA) and E_2_17βG (E1127), E_1_3SO_4_ (E9145), and S-methyl GSH (L-γ-glutamyl-S-methyl-L-cysteinyl-glycine) (M4139) were from Sigma (Oakville, ON, Canada). 

### 4.2. Site-Directed Mutagenesis

DNA primers used for mutagenesis were obtained from Integrated DNA Technologies (Coralville, IA, USA) and their sequences are listed in Table S2. Single hMRP1 mutations G392I, Y404A, Y404F, R405A, R405K, N412A, R415A, R415K, K416A, K416R, E422A, E422D, E422R, D430E, F434A, F434Y, and R615E and the double exchange mutations E422R/R615E were introduced by a ‘one step’ polymerase chain reaction (PCR) mutagenesis technique using a Quikchange II XL Kit (200521-5; Agilent Technologies, Santa Clara, CA, USA) using full-length wild-type pcDNA3.1(-)-MRP1 or the single mutant pcDNA3.1(-)-MRP1-R615E as templates as appropriate [14]. Mutant constructs were fully sequenced (The Center for Applied Genomics, Toronto, ON, Canada) to confirm the presence of the desired mutations and the fidelity of all constructs.

### 4.3. Cell Culture, Transfections, and Preparation of Whole Cell Extracts and Membrane Vesicles

SV40-transformed HEK cells were grown in DMEM supplemented with 7.5% fetal bovine serum at 37 °C in 5% CO_2_/95% air and transfected with wild-type and mutant pcDNA3.1(-)-MRP1 expression vectors using Lipofectamine^®^ 2000 (11668-019; Invitrogen, Carlsbad, CA, USA) (DNA:lipofectamine ratio 1:3) as before [14]. Untransfected cells were included as negative controls. Cells were collected by centrifugation and snap-frozen 48 h post-transfection and stored at −80 °C until needed. Extracts were prepared by first resuspending the transfected cells in solubilization buffer [10 mM Tris (pH 7.5), 150 mM NaCl, 1 mM EDTA, 0.5% sodium deoxycholate, 0.1% SDS, 1% Triton X-100] with EDTA-free protease inhibitor cocktail (11836170001; Roche, Mannheim, Germany) and DNaseI (50 µg ml^−1^) (27-0516-01; Amersham Biosciences, Baie d’Urfé, QC, Canada) and incubating on ice for 10–15 min. The suspension was then centrifuged and protein in the supernatant (WCE) was quantified using the *D_C_* Protein Assay (5000112; Bio-Rad, Mississauga, ON, Canada) with bovine serum albumin as a standard. Membrane vesicles were prepared by disrupting slowly thawed transfected cells by argon cavitation (250 psi, 4 °C, 5 min), followed by a low speed centrifugation. The supernatant was centrifuged (100,000× *g*, 1 h, 4 °C) on a 35% sucrose cushion and the membranes at the interface collected, washed and centrifuged again (128,000× *g*, 20 min, 4 °C). After resuspending the pellet in 50 mM Tris (pH 7.4) and 250 mM sucrose, vesicles were formed by repeated (10 times) passage through a 27-gauge needle as before [7,14]. Vesicles were aliquoted and stored at –80 °C until needed. Vesicular protein concentrations were determined using the Bradford method (500-0006; Bio-Rad) with bovine serum albumin as a standard. 

### 4.4. Immunoblotting

Levels of mutant hMRP1 relative to wild-type hMRP1 in WCE and membrane vesicles were determined by immunoblot analysis [14] using mouse mAb QCRL-1 anti-hMRP1 (epitope amino acids ^918^SSYSGDI^924^) [45] (diluted 1:10,000), and mouse anti-α-tubulin (diluted 1:10,000) (T6074; Sigma) or mouse anti-Na^+^/K^+^-ATPase (diluted 1:10,000) (SC-21712; Santa Cruz Biotechnology, Dallas, TX, USA) as protein loading controls followed by incubation with horseradish peroxidase-conjugated goat anti-mouse antibody (diluted 1:10,000) (31430; Thermo Fisher Scientific, Waltham, MA, USA). Antibodies bound to the blot were detected using a Western Blotting Chemiluminescence Reagent Plus Kit (NEL105; Perkin Elmer, Guelph, ON, Canada) and the blot exposed to HyBlot CL autoradiography film (E3018; Denville Scientific, South Plainfield, NJ, USA) for several time periods to obtain signals within the linear dynamic range. Signals on the film were quantified by densitometry using ImageJ software [46] [http://rsb.info.nih.gov/ij/index.html; version 1.53e] and values adjusted if needed, according to the signal of the protein loading control. Mutant hMRP1 levels were then expressed relative to wild-type hMRP1 levels. 

### 4.5. Vesicular Transport Assays

ATP-dependent transport of ^3^H-labeled organic anions by wild-type and mutant hMRP1 was determined using a vesicular transport assay adapted to a 96-well plate format, performed in duplicate [7,14]. Briefly, each assay tube contained 10 mM MgCl_2_, either 4 mM ATP or AMP together with unlabeled and tritiated forms of organic anion substrate according to the following conditions pre-determined to ensure that transport was linear up until or beyond the time indicated: for LTC_4_ uptake, 50 nM LTC_4_, 10 nCi [^3^H]LTC_4_, 2 µg vesicles, 1 min at 23 °C; for E_2_17βG uptake, 400 nM E_2_17βG, 20 nCi [^3^H]E_2_17βG, 2 µg vesicles, 2 min at 37 °C; and for E_1_3SO_4_ uptake, 3 mM S-methyl GSH, 300 nM E_1_3SO_4_, 25 nCi [^3^H]E_1_3SO_4_, 2 µg vesicles, 1 min at 37 °C. Uptake was stopped by diluting the assay mixture in ice-cold Tris-sucrose buffer and the vesicles captured by filtering through a Unifilter GF/B plate using a Packard Filtermate Harvester. After drying overnight, Microscint^TM^-O (6013611; Perkin Elmer) was added to the filter plate to allow the tritium to be quantified. ATP-dependent uptake of organic anions was calculated by subtracting uptake of ^3^H-labeled substrate by hMRP1-enriched vesicles in the presence of AMP from uptake in the presence of ATP. If needed, uptake levels were adjusted to take into account the differences in levels of wild-type and mutant hMRP1 protein in the vesicles as determined by a companion immunoblot, and then mutant hMRP1 uptake activity was expressed as a percent of wild-type hMRP1 activity. 

### 4.6. Statistics

When three or more independent experiments were performed, data were analyzed for differences using an unpaired t-test performed using GraphPad Prism^TM^ (GraphPad Software, La Jolla, CA, USA) with a significance threshold of *p* < 0.05. 

## 5. Conclusions

CL4 contains seven (and possibly more) amino acids crucial for hMRP1 expression further establishing the importance of the structural integrity of this first intracellular loop of MSD1. Somewhat unexpectedly, the seven mutation-sensitive residues thus far identified lie outside the CL4 coupling helix that interfaces with the degenerate nucleotide binding site of the transporter. While many of these mutation-sensitive amino acids are ionizable, they also include two aromatic residues and for the first time in hMRP1, a glycine residue. The mechanism(s) underlying the decreased levels of the multiple CL4 mutants described here and previously remain unclear [19,23]. Thus far, exchange mutagenesis experiments, even when guided by both structural and homology-based models of hMRP1, have not provided decisive insights. In addition, a high degree of conservation is not a reliable indicator of the importance of a CL4 amino acid for hMRP1 expression because non-conservative substitutions of the upstream Arg^394^ and Lys^396^ and downstream Arg^433^ and Asp^436^ had no adverse effect on hMRP1 levels [22,23] even though these residues are just as conserved as those studied here. Additional structures that capture the different stages of hMRP1 biogenesis, together with strategic mutagenesis and modeling studies using molecular dynamics simulations, are needed to elucidate the interdomain and intradomain bonding interactions involved in its assembly into a stably folded, active transporter [47,48,49].

## Figures and Tables

**Table 1:** Mutations of Asn^412^, Arg^415^, and Lys^416^ within the CL4 coupling helix have no deleterious effect on hMRP1 protein expression levels. Wild-type and mutant hMRP1 expression levels were measured by immunoblotting of WCE (10 µg) and membrane vesicles (MV) (1 µg) prepared from transfected HEK cells. hMRP1 levels were estimated using densitometry, values normalized according to the signal of the α-tubulin loading control (for WCE) or the signal of the Na^+^/K^+^-ATPase loading control (for MV), and expressed as a percent of wild-type (WT) hMRP1 levels. Values represent the means (±SD) of results obtained from 3–5 independent transfections (*n*).

Mutant	hMRP1 Levels (% WT-hMRP1) *(n)*	
	WCE	MV
N412A	133 ± 15 *(3)*	119 ± 28 *(3)*
R415A	83 ± 16 *(5)*	81 ± 31 *(3)*
R415K	104 ± 21 *(3)*	85 ± 26 *(4)*
K416A	103 ± 21 *(3)*	97 ± 39 *(3)*
K416R	138 ± 12 *(3)*	127 ± 20 *(3)*

**Table 2:** Effect of conservative substitutions of CL4 residues outside the coupling helix on organic anion transport by hMRP1. Transport activity was measured as ATP-dependent uptake of [^3^H]LTC_4_, [^3^H]E_2_17βG and [^3^H]E_1_3SO_4_ (in the presence of 3 mM S-methyl GSH (S-Me GSH)) into inside-out membrane vesicles prepared from transfected cells and expressed as a percent of wild-type (WT) hMRP1 uptake. The values shown have been adjusted to take into account minor differences in mutant hMRP1 levels in the membrane vesicles relative to wild-type MRP1. Values shown represent the means (±SD) of results obtained from three independent experiments.

Mutant.	Transport Activity (% WT-hMRP1)		
	LTC_4_	E_2_17βG	E_1_3SO_4_ (+ S-Me GSH)
HEK ctrl	6 ± 4 *	0 ± 0 *	7 ± 2 *
WT-hMRP1	100 ± 10	100 ± 10	100 ± 15
Y404F	90 ± 2	102 ± 9	107 ± 17
E422D	84 ± 16	109 ± 9	89 ± 14
D430E	87 ± 11	102 ± 4	104 ± 18

* Significantly different from wild-type hMRP1 (*p* < 0.05).

## Data Availability

All relevant data for this study are included in the manuscript and its Supplementary Files.

## Supplementary Materials

The following are available online at https://www.mdpi.com/article/10.3390/ijms22189710/s1.
