# Supplementary material for: The First Cytoplasmic Loop in the Core Structure of the ABCC1 (Multidrug Resistance Protein 1; MRP1) Transporter Contains Multiple Amino Acids Essential for Its Expression
Source: Int J Mol Sci. 2021 Sep 8;22(18):9710. doi: 10.3390/ijms22189710 (PMC8469891; doi:10.3390/ijms22189710)
Supplement: Supplementary file 1 [file ijms-22-09710-s001.zip › ijms-1351362-supplementary.pdf]

Article

The First Cytoplasmic Loop in the Core Structure of the ABCC1 (Multidrug Resistance Protein 1; MRP1) Transporter Contains Multiple Amino Acids Essential for Its Expression

Gwenaëlle Conseil [1,*] and Susan P. C. Cole [1,2]

1 Division of Cancer Biology and Genetics, Queen's University Cancer Research Institute, Kingston, ON K7L 3N6, Canada; spc.cole@queensu.ca
2 Department of Pathology & Molecular Medicine, Queen's University, Kingston, ON K7L 3N6, Canada; spc.cole@queensu.ca
* Correspondence: conseilg@queensu.ca; Tel.: +1-613-533-6358

Supplementary Figures & Tables

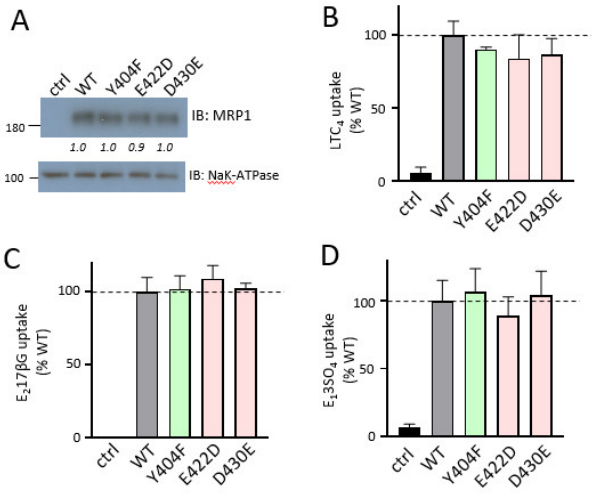

Figure S1. Conservative substitutions of CL4 Tyr^{404}, Glu^{422} and Asp^{430} outside coupling helix have no effect on ATP-dependent organic anion transport by hMRP1. (**A**) Shown is a representative immunoblot of inside-out membrane vesicles (1 μg protein per lane) prepared from HEK cells transfected with wild-type (WT) and Y404F, E422D, and D430E mutant hMRP1 pcDNA expression vectors as well as untransfected cells (negative control) (ctrl). The blot was probed with anti-hMRP1 (Mab QCRL-1) and anti-Na^+/K^+-ATPase as a membrane protein loading control. The images in each panel are from a single blot with the region between hMRP1 (1 sec exposure) and Na^+/K^+-ATPase (2 sec exposure) cropped out. Molecular weight markers (kDa) are to the left. Italicized numbers between the 2 panels indicate mutant hMRP1 levels relative to wild-type hMRP1 after correcting for levels of the protein loading control as measured by densitometry **(B–D)** Transport activity was measured as ATP-dependent uptake of **(B)** [^{3}H]LTC_4, **(C)** [^{3}H]$E_2$17βG and **(D)** [^{3}H]$E_1$3SO_4 (in the presence of 3 mM S-MeGSH) into inside-out membrane vesicles and expressed as a percent of wild-type hMRP1 uptake. The values shown have been adjusted to take into account minor differences in mutant hMRP1 levels in the membrane vesicles relative to wild-type (WT) hMRP1. Bars represent the mean values (± SD) of results obtained from three independent experiments from at least two membrane vesicle preparations. $p > 0.05$.

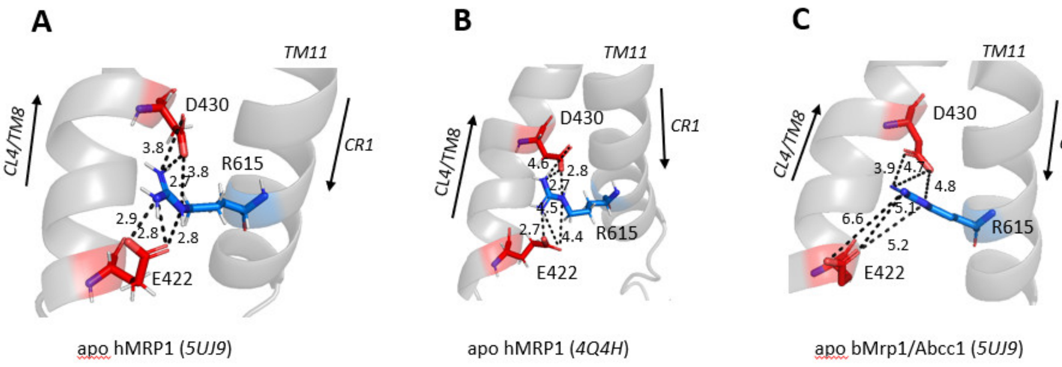

Figure S2. Interatomic distances among the ionizable side chains of CR1-Arg615, CL4-Asp430 and CL4-Glu422 in models of hMRP1 and bMrp1/Abcc1. Models showing the spatial relationships of the side chains of CL4-Glu422 and CL4-Asp430 with CR1-Arg615 (**A** and **B**) in the atomic homology models of hMRP1 based on (A) apo bMrp1/Abcc1 [PDB: 5UJ9, 3.49Å] and (**B**) apo TM287/288 [PDB: 4Q4H, 2.53Å]; (C) in the cryo-EM structure of apo bMrp1/Abcc1 [PDB: 5UJ9, 3.49Å]. Interatomic distances (Å) are shown and indicate possible electrostatic bonding networks among the three ionizable side chains for the hMRP1 models ('sandwich') (charged centers of multiple contributing atoms ≤4Å) (**A** and **B**) but not for the bMrp1/Abcc1 structure (**C**).

Table S1. Predicted interatomic distances between charged centers of CL4-Arg405 and CL7-Asp1179, and CL4-Glu422 and CR1-Arg615. aThe distances between all combinations of charged atoms of ion pairs Arg405/Asp1179 and Glu422/Arg615 were estimated using the PyMol measuring tool. Distances were measured between the three positively charged atoms of the guanidinium group of Arg405 and Arg615 (NH1, NH2 and NE) and the two oxygen atoms of the α-carboxylic acid group of Asp1179 (OD1 and OD2) and Glu422 (OE1 and OE2). Those distances meeting the criteria for a salt bridge or ion pair (i.e. ≤4Å) are underlined and in boldface.

		**Structure/Model (PDB)**		
		**apo bAbcc1 (5UJ9)**	**apo hMRP1 (5UJ9)**	apo hMRP1 (4Q4H)
			Interatomic distances (Å)[a]	
R405/D1179	OD1-NH1	**3.6**	**3.9**	9.2
	OD1-NH2	4.5	**2.8**	11.2
	OD2-NH1	4.1	**2.8**	7.1
	OD2-NH2	**3.8**	**2.9**	9.0
	OD1-NE	5.7	5.0	11.3
	OD2-NE	5.6	4.6	9.2
E422/R615	OE1-NH1	4.7	4.4	4.5
	OE1-NH2	5.1	**2.8**	6.1
	OE2-NH1	6.4	4.7	**2.7**
	OE2-NH2	6.6	**2.9**	4.8
	OE1-NE	5.2	**2.8**	4.4
	OE2-NE	7.2	4.1	**3.7**

Table S2. Sequences of mutagenesis primers used to create the hMRP1 mutants in this work. Substituted nucleotides are underlined.

	hMRP1 Mutant	Sense Primer Sequence
upstream flank	G392I	5' CAC ATC TGC TTC GTC AGT ATC ATG AGG ATC AAG ACC GC 3'
	Y404A	5' GTC ATT GGG GCT GTC GCT CGG AAG GCC CTG GT 3'
	Y404F	5' GCT GTC ATT GGG GCT GTC TTT CGG AAG GCC 3'
	R405A	5' T GTC ATT GGG GCT GTC TAT GCG AAG GCC CTG G 3'
	R405K	5' GCT GTC ATT GGG GCT GTC TAT AAG AAG GCC CTG GT 3'
coupling helix region	N412A	5' AAG GCC CTG GTG ATC ACC GCT TCA GCC AGA AAA TCC TC 3'
	R415A	5' GTG ATC ACC AAT TCA GCC GCA AAA TCC TCC ACG GTC GG 3'
	R415K	5' G ATC ACC AAT TCA GCC AAG AAA TCC TCC ACG GTC G 3'
	K416A	5' C ACC AAT TCA GCC AGA GCA TCC TCC ACG GTC GGG 3'
	K416R	5' GTG ATC ACC AAT TCA GCC AGA AGA TCC TCC ACG GT 3'
downstream flank	E422A	5' AA TCC TCC ACG GTC GGG GCG ATT GTC AAC CTC 3'
	E422D	5' AA TCC TCC ACG GTC GGG GAT ATT GTC AAC CTC 3'
	E422R	5' AA TCC TCC ACG GTC GGG CGG ATT GTC AAC CTC 3'
	D430E	5' C AAC CTC ATG TCT GTG GAG GCT CAG AGG TTC 3'
	F434A	5' GTG GAC GCT CAG AGG GCC ATG GAC TTG GCC AC 3'
	F434Y	5' GTG GAC GCT CAG AGG TAT ATG GAC TTG GCC ACG T 3'
CR1	R615E	5' CG AGT GTC TCC CTC AAA GAA CTG AGG ATC TTT CTC TCC 3'
